# Analysis of National Institutes of Health Funding for the COVID-19 Pandemic

**DOI:** 10.1093/ofid/ofae064

**Published:** 2024-03-26

**Authors:** Adishesh K Narahari, Taylor M Horgan, Anirudha S Chandrabhatla, D Chris Gist, Paranjay D Patel, Mark A Lantieri, Jeffrey M Sturek, Claire L Davis, Patrick E H Jackson, Taison D Bell

**Affiliations:** Division of Cardiothoracic Surgery, University of Virginia School of Medicine, Charlottesville, Virginia, USA; School of Medicine, University of Virginia, Charlottesville, Virginia, USA; School of Medicine, University of Virginia, Charlottesville, Virginia, USA; School of Medicine, University of Virginia, Charlottesville, Virginia, USA; Department of Cardiovascular Surgery, Houston Methodist Hospital, Houston, Texas, USA; Department of Orthopedic Surgery, Johns Hopkins Hospital, Baltimore, Maryland, USA; School of Medicine, University of Virginia, Charlottesville, Virginia, USA; Division Of Pulmonary and Critical Care Medicine, University of Virginia, Charlottesville, Virginia, USA; School of Medicine, University of Virginia, Charlottesville, Virginia, USA; Division Of Pulmonary and Critical Care Medicine, University of Virginia, Charlottesville, Virginia, USA; School of Medicine, University of Virginia, Charlottesville, Virginia, USA; Division of Infectious Diseases and International Health, University of Virginia, Charlottesville, Virginia, USA; School of Medicine, University of Virginia, Charlottesville, Virginia, USA; Division Of Pulmonary and Critical Care Medicine, University of Virginia, Charlottesville, Virginia, USA; Division of Infectious Diseases and International Health, University of Virginia, Charlottesville, Virginia, USA

**Keywords:** clinical trials, COVID-19, grants, NIH funding, SARS CoV-2

## Abstract

**Background:**

Evaluating the National Institute’s Health’s (NIH's) response to the coronavirus disease 2019 (COVID-19) pandemic via grants and clinical trials is crucial to determining the impact they had on aiding US citizens. We determined how the NIH's funding for COVID-19 research was disbursed and used by various institutions across the United States.

**Methods:**

We queried NIH RePORTER and isolated COVID-19–related grants from January 2020 to December 2021. We analyzed grant type, geographical location, and awardee institution. Manuscripts published from these grants were quantitatively analyzed. COVID-19 clinical trials were mapped and distances from counties to clinical trial sites were calculated using ArcGis.

**Results:**

A total of 2401 COVID-19 NIH grants resulted in 14 654 manuscripts from $4.2 billion and generated more than 150 000 citations. R01s make up 32% of grants (763/2401) and 8% of funding ($329 million). UM1 grants account for the majority of funding (30.8%; $1.3 Billion). Five states received 50.6% of funding: North Carolina, Washington, New York, California, and Massachusetts. Finally, of the 1806 clinical trials across 1266 sites in the United States, the majority were in metropolitan areas in close proximity to areas of high COVID-19 disease burden.

**Conclusions and Relevance:**

Evaluating the outcome of the NIH's response to the COVID-19 pandemic is of interest to the general public. The present study finds that the NIH disbursed more than $4 billion in funding to large consortiums and clinical trials to develop diagnostics, therapeutics, and vaccines. Approximately 8% of funding was used for R01 grants. Clinical trial sites were generally located in areas of high COVID-19 burden.

Funding from the National Institutes of Health (NIH) is an important driver of biomedical research in the United States. This financial support is often in the form of study section grants to individual research projects [1]. NIH support, particularly through the National Institute of Allergy and Infectious Diseases, has helped lead the response to many novel epidemics and pandemics since its inception, including the global HIV/acquired immune deficiency syndrome pandemic, the 2002–2004 severe acute respiratory coronavirus (SARS-CoV)-1 outbreak, the 2009 H1N1 influenza virus pandemic, and the 2014–2016 Ebola virus outbreak, among many others [2–4]. This funding has helped contribute to our understanding of virus development, pathophysiology and virulence, infection complications, and vaccines and other treatments, and has ranged from supporting individual studies to entire research departments and study sections [3, 5–7].

However, the NIH response to the 2019 SARS-CoV-2 pandemic was unique in that a predominant source of NIH funding came from the Coronavirus Aid, Relief, and Economic Security (CARES) Act from the US Congress. In addition to payments to individuals and small business loans, the CARES Act provided nearly $1 billion in supplemental NIH funding for studies investigating SARS-CoV-2 [8]. In this study, we examined the use of this additional funding, including an analysis of where and how funds were spent.

## METHODS

### Grant and Manuscript Data Collection

The NIH's Research Portfolio Online Reporting Tools Expenditures and Results (RePORTER) database was queried to collect grant information [9]. NIH RePORTER contains grant information including principal investigator, institution where research will be performed, funding, supported publications, and relevant patent information. Per the instructions on the NIH website, COVID-specific grants were identified using the “NIH COVID-19 Response” filter. A Python script using Phantom JS and BeautifulSoup was used to collect the following information for each grant of interest: identifier number, title, type of grant (eg, R01), principal investigator, awardee organization, awardee department, grant start date, grant end date, awarding institute, awarding study section, total funding amount, number of publications, PubMed Identification of each publication, and the journal of each publication. We used the NIH iCite tool to determine the citation count and the BioC PubMed API to collect the title and abstract for each publication [10]. All information was gathered in December 2021. Grants were collected by the filtering available on the NIH RePORTER website for COVID-focused grants.

## Supplementary Material

ofae064_Supplementary_Data

## Figures and Tables

**Figure 1.**
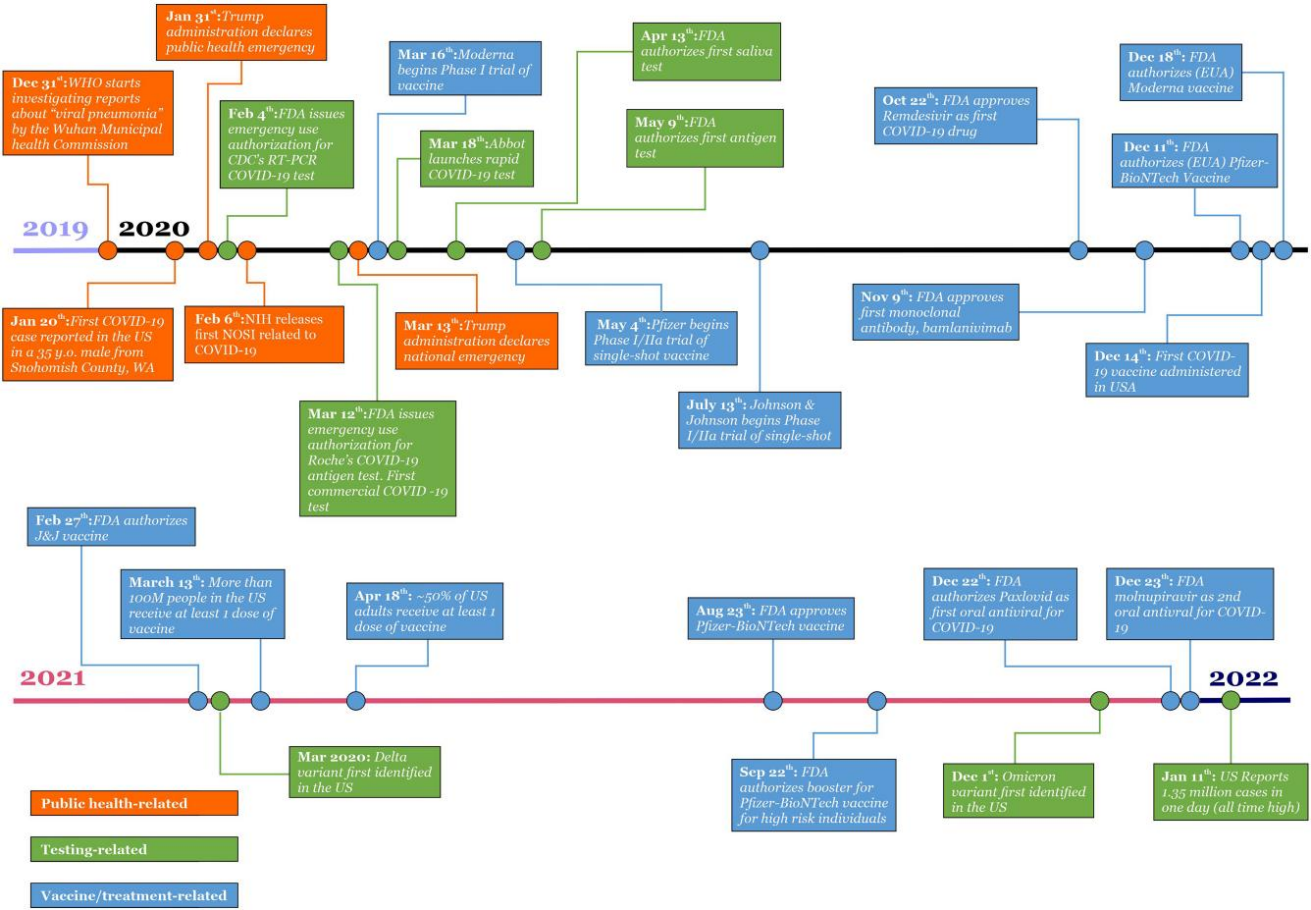
Timeline of COVID-19 events.

**Figure 2.**
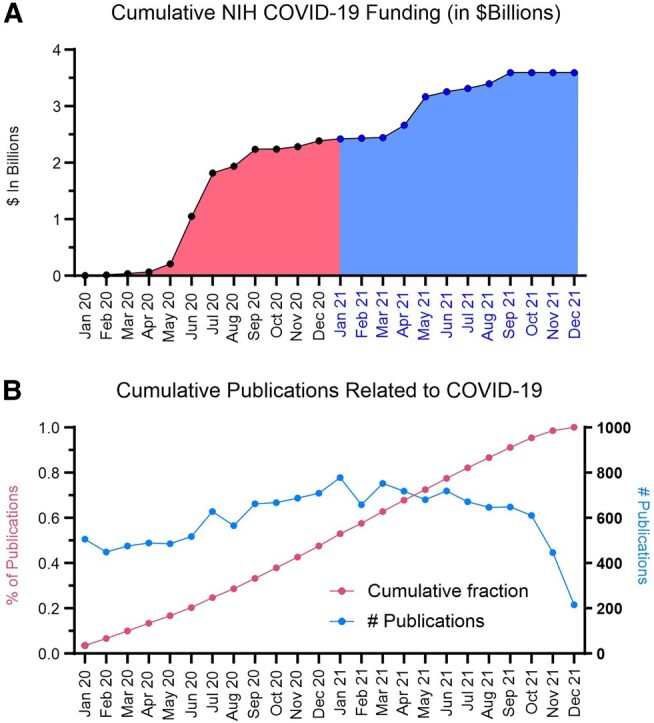
Timeline of NIH COVID-19 funding disbursement and publication of COVID-19 manuscripts. *A*, Project start dates of COVID-19 grants were used and binned by month from January 2020 to December 2021. Totals from the awards of grants from that month are shown on the y-axis in $Billions. *B*, Timeline of COVID-19 publications from January 2020 to December 2021. The raw number of publications is shown on the y-axis on right side and the cumulative percentage of publications is shown on the y-axis on left side.

**Table 1.** Grant Type Analysis of COVID-19 Funding

Grant Type	Total Funding (% of Total)	Mean Funding per Grant (SD)	Number of Grants (% of Total)	Total Number of Publications (Mean per Grant [SD])	Total Number of Citations (Mean per Grant [SD])
UM1	$1 302 120 048 (30.8%)	$8 680 800 ($38 203 771)	150 (6.2%)	849 (5.7 [14.6])	16 480 (110 [512])
OT2	$1 065 866 609 (25.3%)	$26 646 665 ($76 718 309)	40 (1.7%)	13 (0.3 [1.5])	200 (5 [32])
U54	$343 623 962 (8.1%)	$2 623 083 ($8 471 865)	131 (5.5%)	1417 (10.8 [20.1])	7768 (59 [144])
R01	$328 750 109 (7.8%)	$430 865 ($404 470)	763 (31.8%)	3202 (4.2 [7.0])	50 945 (67 [321])
U24	$218 279 178 (5.2%)	$5 197 124 ($14 139 704)	42 (1.7%)	408 (9.7 [26.1])	2745 (65 [166])
U01	$165 085 872 (3.9%)	$767 841 ($1 065 361)	215 (8.9%)	1070 (5.0 [10.0])	7429 (35 [84])
U19	$157 631 096 (3.7%)	$1 185 196 ($2 516 097)	133 (5.5%)	961 (7.2 [13.7])	25 264 (190 [640])
UL1	$157 775 942 (3.6%)	$2 023 679 ($4 778 535)	75 (3.1%)	1822 (24.3 [46.4])	10 822 (144 [381])
P01	$51 255 733 (1.2%)	$1 385 290 ($3 478 800)	37 (1.5%)	449 (12.1 [16.3])	7120 (192 [515])
Total	$4 216 603 887	$1 756 296 ($14 505 288)	2401	14 654	159 902

Abbreviation: SD, standard deviation.

**Figure 3.**
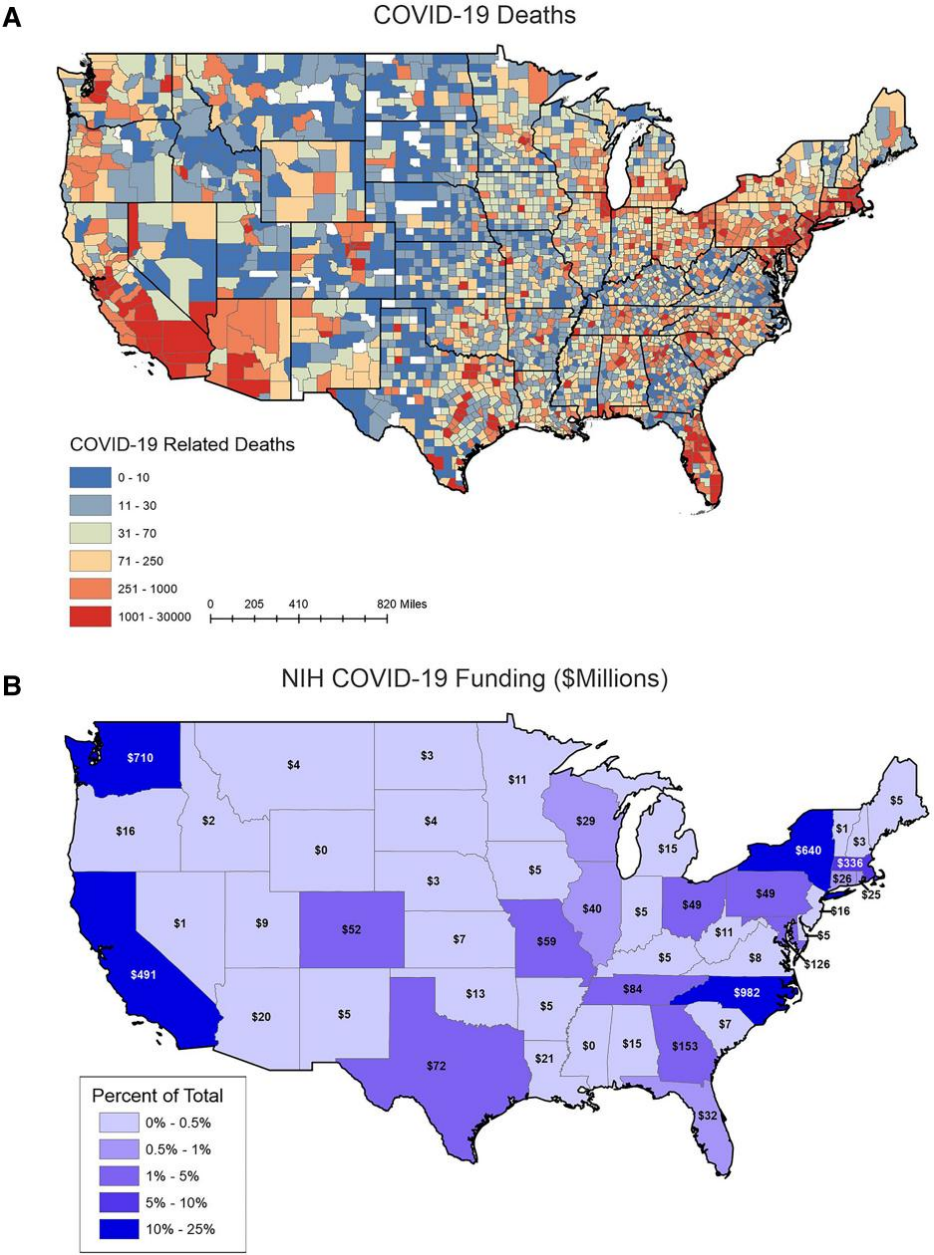
Heatmaps of COVID-19 deaths and COVID-19 NIH funding. *A*, COVID-19 death tolls for each county. The lowest death tolls (0–10) are shown in dark blue and the highest death tolls are shown in dark red (1001–30 000). *B*, COVID-19 funding for each state from January 2020 to December 2021.

**Table 2.** Top 5 Institutions Receiving COVID-19 NIH Funding

Institution	Project Description	Funding
**Fred Hutchinson Cancer Research Center**	-	$624 705 137.00
HVTN 405/HPTN 1901 Characterizing SARS-CoV-2–specific Immunity in Convalescent Individuals	A clinical trial (observational cohort study) intended to enroll 400 participants to study the serology in 3 cohorts: hospitalized (vs non), symptomatic (vs non), and severe infection (regardless of hospitalization) to understand adaptive immune responses to SARS-CoV-2. Other aims include creation of immunologic assays to interrogate immune response, identification of serological markers differentiating infection from vaccination, and preparation for future COVID-19 vaccine trials.	$394 355 502.00
CoVPN 3005—Efficacy, Immunogenicity, and Safety of SARS-CoV-2 Recombinant Protein Vaccine With Adjuvant in Adults 18 Years of Age and Older	Phase 3 clinical trial (modified double-blind, placebo-controlled) testing efficacy, safety, and immunogenicity of SARS-CoV-2 recombinant protein adjuvanted with AS03 (both monovalent and bivalent) vaccines in naïve adult participants. Specifically, the trial is measuring the incidence of symptomatic COVID-19 > 14 d after participants receive the second vaccine dose.	$61 429 109.00
Personal Protective Equipment for Resources for COVID-19–related Vaccine and Treatment Clinical Trials and Clinical Studies	Funding for adequate PPE in the conduction of COVID-19 vaccine trials.	$46 263 849.00
**New York University School of Medicine**	-	$511 653 095.00
OTA-21-015A Post-Acute Sequelae of SARS-CoV-2 Infection Initiative: NYU Langone Health Clinical Science Core, Data Resource Core, and PASC Biorepository Core	An initiative seeking greater understanding of recovery from SARS-CoV-2; specifically, after acute infection and from postacute sequalae of SARS-CoV-2 (PASC). Specifically, the underlying biology, clinical spectrum, incidence/prevalence, and effect of treatment on recovery.	$448 259 603.00
Clinical and Translational Science Award	…	$32 119 186.00
Establishment of the New York University Vaccine and Treatment Evaluation Unit (NYU VTEU)	A phase 3 randomized, double-blind, placebo-controlled trial administered to 30 000 persons studying efficacy, safety, and immunogenicity of AZD1222, a recombinant adenovirus expressing the SARS-CoV-2 spike (S) surface glycoprotein in the prevention of COVID-19. Additionally, a separate trial is studying the efficacy and safety of anti-spike SARS-CoV-2 monoclonal antibodies in the prevention of infection in household contacts of infected individuals.	$16 035 211.00
**Research Triangle Institute**	-	$501 968 796.00
ACTIV Integration of Host-targeting Therapies for COVID-19 Administrative Coordinating Center	Phase 2 and 3 randomized, controlled clinical trials studying host-targeting treatments to prevent and treat SARS-CoV-2 with particular focus on preventing cardiovascular, pulmonary, and hematologic complications of COVID-19. Additional objectives include identifying prognostic biomarkers, improving clinical care of COVID-19 patients, and slowing or preventing COVID-19 progression.	$498 112 068.00
HEAL Initiative: Antenatal Opioid Exposure Longitudinal Study Consortium	Studying the effect of antenatal opioid exposure on infant brain development via serial magnetic resonance imaging.	$1 750 001.00
Genomic Resource Grant for the PhenX Toolkit—expansion and sustainability PhenX Supplement for COVID-19 Research	Expansion of PhenX Toolkit, a bioinformatics tool, to aid in COVID-19 data collection and collaborative research.	$955 000.00
**University of California Los Angeles**	-	$289 088 327.00
Leadership and Operations Center (LOC), AIDS Clinical Trials Group (ACTG); LOC 1/	Funding for the construction of COVID-19 clinical trial pods to reduce exposure of COVID-19 to those with HIV.	$181 942 638.00
AIDS Clinical Trials Group for Research on Therapeutics for HIV and Related Infections	Studies aiming to reduce morbidity and mortality in patients with HIV/AIDS, tuberculosis, and/or hepatitis B.	$60 731 236.00
CoVPN 3502/ACTIV-2/A5401	A phase 3, randomized, double-blind, placebo-controlled trial studying efficacy and safety of anti-spike SARS-CoV-2 monoclonal antibodies in the prevention of COVID-19 transmission via household contact.	$19 807 516.00
**Duke University**	…	$212 122 911.00
ACTIV-6	Funding of ACTIV-6, a master clinical trial protocol studying repurposed drugs for the treatment of COVID-19.	$115 543 799.00
RADx-UP CDCC	Funding RADx-UP, a program coordinating projects studying SARS-CoV-2 infection in underserved and/or vulnerable populations to increase access to and effectiveness of diagnostic methods.	$56 405 724.00
Design and Development of a Pan-betacoronavirus Vaccine	Funding for the design and development of pan-betacoronavirus vaccines to prevent future beta-CoV human outbreaks.	$17 521 953.00

The top 3 grants from each institution are displayed.

Abbreviations: CoV, coronavirus; HIV, human immunodeficiency virus; PPE, personal protective equipment.

**Figure 4.**
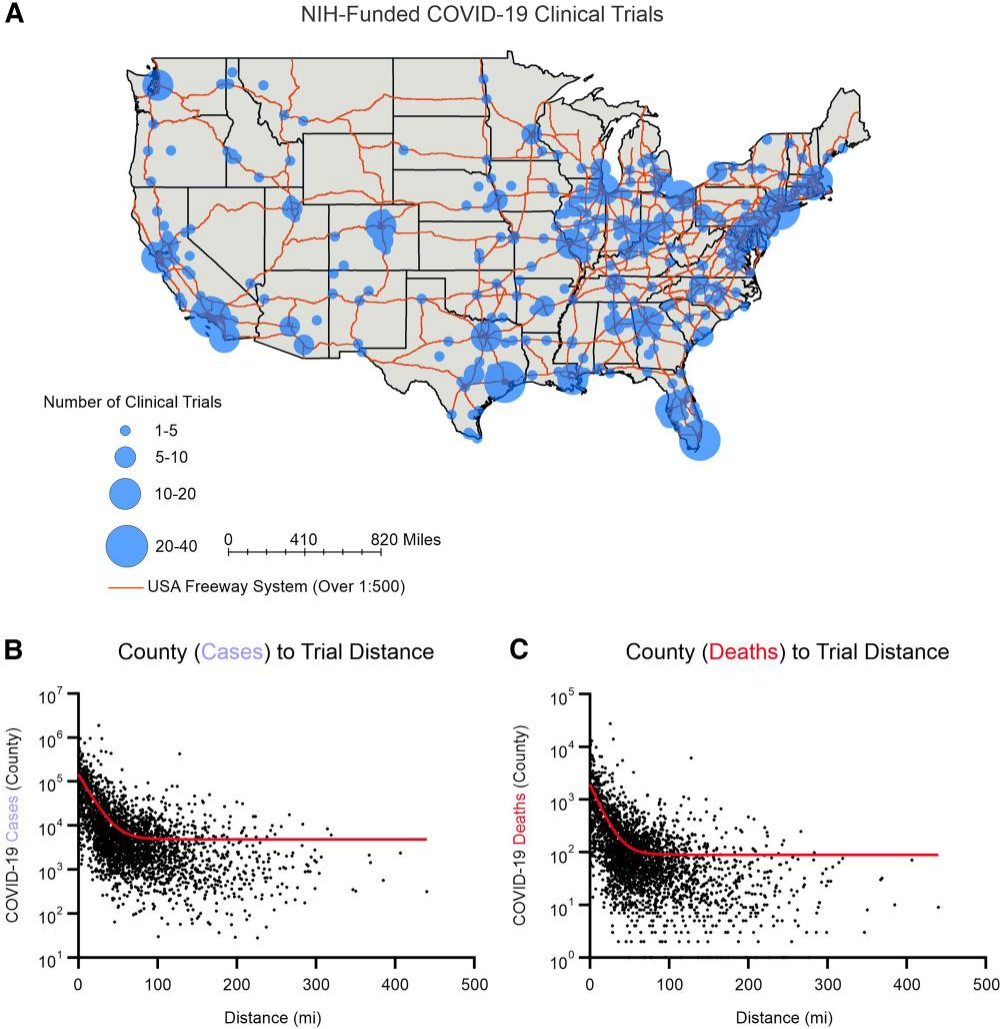
COVID-19 clinical trials. *A*, COVID-19 clinical trials shown by density of clinical trials in each location. *B* and *C*, Distance from the center of each county to the nearest clinical trial site based is plotted against the number of COVID-19 cases or deaths in the respective county.

## Analysis of COVID-19 Deaths

Data from *USAFacts* were used to develop a map of the COVID-19 deaths in the United States by county. *USAFacts* is a not-for-profit organization that is one of the largest repositories of US government data [12]. Since the beginning of the COVID-19 pandemic, *USAFacts* has aggregated data from the Centers for Disease Control and state and local health agencies to report key metrics such as cases, deaths, and vaccination progress. Data on county-level COVID-19 deaths from *USAFacts* were matched to county Federal Information Processing System codes.

## RESULTS

### Institutional Analysis

While analyzing large grants (>$200 million) that were awarded, we noticed that a few institutes in particular received a majority of funding. Fred Hutchinson Cancer Research Center, New York University School of Medicine, Research Triangle Institute, University of California Los Angeles, and Duke University collectively received a total of $2.14 billion, or approximately 50.6% of all COVID-19 funding (Table 2). The top 3 grants at each institution are reported. The largest grant awarded during the COVID-19 pandemic was “HVTN 405/HPTN 1901 Characterizing SARS-CoV-2-specific immunity in convalescent individuals.” This grant was for approximately $400 million to study the serology of patients with SARS-CoV-2 and their immune response to the infection. Furthermore, the grant was also awarded for the identification of markers to study infection versus immunization, and to prepare for future COVID-19 vaccine trials. We have included an in-depth analysis on the top 20 grants related to COVID-19 (Supplementary Table 2). Here, we describe the number of publications and citations that have resulted from the grants as well as linked this information to the clinical trials they have helped to fund to understand the NIH's real-world impact.

## DISCUSSION

As the largest biomedical funding entity in the United States, NIH’s role is crucial in the research response to epidemics and pandemics. The NIH disbursed more than $4 billion in funding earmarked for the study of SARS-CoV-2, the COVID-19 pandemic, and for the development of therapeutics between January 2020 and December 2021 [8]. The rapid mobilization in funds was unprecedented and made possible by the CARES Act passed by the US government [14]. In this study, we describe the distribution, utilization, and results of this funding.

Institutions with the infrastructure for large scale genomics, infectious disease research, and ability to perform clinical trials were funded highly. Fifteen large grants to 5 institutions accounted for more than half of NIH COVID-19 research funding. These grants were awarded to institutions that were poised to perform large-scale research. A preference for larger grants requiring large collaborations were funded as a potential means to recruit large numbers/groups of researchers to develop solutions in a multidisciplinary approach. Less funding was allocated to independent investigators for Research Project Grants.

Although large grants are instrumental in clinical research, R01 grants are the mainstay of research projects at the single principal investigator/laboratory level [15]. The majority of funding in our study has been from large consortium grants. This suggests that the primary means of funding was not through investigator driven, R01-funded projects, but projects intended to drive COVID-19 research at a much larger scale. These large grants have allowed for investigation of the new virus using a multimodal approach. At the same time, these grants have helped fund clinical trials for infection testing and vaccine development, all critical necessities during a pandemic. Although large consortium grants can accelerate the progress of obvious priorities, they may not be as efficient in generating scientific breakthroughs through creative individual investigator-driven efforts.

Accessibility to health care, especially in a pandemic, is incredibly important [16, 17]. Clinical trial sites for COVID-19 were generally located in areas of high COVID cases and death tolls (Figure 4). However, some large counties with a high burden of disease were not served by clinical trial sites. A cause for these outlier counties is not readily apparent. They may be areas that stochastically and unintentionally were located further from trial sites compared with their neighboring counties. Some of these counties are quite populous and are not necessarily health care deserts (ie, have health care infrastructure). This result warrants further analysis to ensure that access to clinical trials is equitably and efficiently distributed.

The long standing and domino effects of the NIH's $4 billion investment into COVID-19 is extremely difficult to quantify. For example, Watson et al. predict that 14.4 million lives were saved because of COVID-19 vaccines [18]. Surely, the NIH's investment leading to basic science insights, vaccine development, and clinical trial support contributed to this astounding achievement. However, direct contribution of dollars to lives saved is difficult to make.

Our study had a few limitations. First, we only studied the grants given by the COVID-19–related funding on NIH RePORTER. If a grant was not included on this list as dictated by the NIH, we did not include it in our analysis. We were limited by the accuracy of the data given for each grant in NIH RePORTER (eg, funding, citations) and in iCite, the tool used for calculating the number of citations for each manuscript. Both of these databases are maintained by the NIH. Additionally, grants with more than 15 pages of citations were not included in our analysis because of technical limitations with our script. Finally, in the cases of multisite trials and research networks, the funds are often then distributed in the form of subawards to other institutions within these large networks and this allocation is not captured in our analysis.

During the ongoing COVID-19 pandemic, the importance of understanding SARS-CoV-2 and developing therapies/vaccines cannot be understated. To this end, COVID-19 funding via the CARES Act has been distributed across the United States for use in research and in clinical trials. Here, we show the research productivity and rapid response to the disbursement of those funds by researchers all across the country. Funding was released in 2 waves for a total of $4.2 billion. A majority of these funds were to large consortia that were established to pursue key diagnostic and therapeutic priorities. These funds were crucial to driving COVID-19–related publications. Finally, we also show that COVID-19–related clinical trials sites are in areas of high COVID-19 disease burden.

## Supplementary Data

Supplementary materials are available at *Open Forum Infectious Diseases* online. Consisting of data provided by the authors to benefit the reader, the posted materials are not copyedited and are the sole responsibility of the authors, so questions or comments should be addressed to the corresponding author.
