# Supplementary material for: Analysis of National Institutes of Health Funding for the COVID-19 Pandemic
Source: Open Forum Infect Dis. 2024 Mar 26;11(3):ofae064. doi: 10.1093/ofid/ofae064 (PMC10965119; doi:10.1093/ofid/ofae064)
Supplement: ofae064_Supplementary_Data [file ofae064_supplementary_data.zip › Supplemental Table 1.docx]

|  | **State** | **Funding ($)** | **COVID Deaths (as of 1/10/22)** | **Funding per COVID death** |
| --- | --- | --- | --- | --- |
| **Top 5 States** | Washington | 710,415,691 | 10,028 | 70,843.21 |
|  | North Carolina | 982,284,865 | 19,685 | 49,900.17 |
|  | Massachusetts | 336,592,135 | 20,643 | 16,305.39 |
|  | New York | 639,628,285 | 60,460 | 10,579.36 |
|  | Maryland | 126,053,436 | 12,258 | 10,283.36 |
| **Bottom 5 States*** | Virginia | 7,764,286 | 15,671 | 495.46 |
|  | South Carolina | 6,573,676 | 14,793 | 444.38 |
|  | Kentucky | 5,267,758 | 12,425 | 423.96 |
|  | Indiana | 4,561,747 | 19,796 | 230.44 |
|  | Nevada | 720,000 | 8,528 | 84.43 |

*Excluding Mississippi and Wyoming as both states did not receive any funding
