# Supplementary material for: Analysis of National Institutes of Health Funding for the COVID-19 Pandemic
Source: Open Forum Infect Dis. 2024 Mar 26;11(3):ofae064. doi: 10.1093/ofid/ofae064 (PMC10965119; doi:10.1093/ofid/ofae064)
Supplement: ofae064_Supplementary_Data [file ofae064_supplementary_data.zip › Supplemental Table 2.docx]

| **Grant number** | **Grant titles** | **Funding ($)** | **# of publications & citations** | **Citations / $100k funding** | **Clinical trials** |
| --- | --- | --- | --- | --- | --- |
| UM1AI068614 | - HVTN 405/HPTN 1901 Characterizing SARS-CoV-2-specific immunity in convalescent individuals - CoVPN 3005 - Efficacy, Immunogenicity, and Safety of SARS-CoV-2 Recombinant Protein Vaccine with Adjuvant in Adults 18 Years of Age and Older - HIV Vaccines Clinical Trials Network Leadership and Operations Center | 498,507,624 | 10 & 280 | 0.06 | - NCT05359250: Myocardial Injury and Dysfunction Associated With COVID-19 Vaccination - NCT05074719: Index Individuals in SARS-CoV-2 Prevention Research Studies - NCT05182125: Evaluating the Safety and Immunogenicity of HIV-1 Vaccines Based on Chimpanzee Serotypes of Ad Expressing Clade C gp140 and a CH505TF gp120 Protein Boost in Healthy, HIV- Uninfected Adult Participants - NCT04607408: Evaluating Safety and Immune Response to the HIV-1 CH505 Transmitted/​Founder gp120 Adjuvanted With GLA-SE in Healthy, HIV-exposed Uninfected Infants - NCT05168813: Efficacy Study of COVID-19 mRNA Vaccine in Regions With SARS-CoV-2 Variants of Concern - NCT04403880: Characterizing SARS-CoV-2-specific Immunity in Individuals Who Have Recovered From COVID-19 - NCT04860323: Analytical Treatment Interruption (ATI) to Assess the Immune System's Ability to Control HIV in Participants Who Became HIV-infected During the HVTN 703/​HPTN 081 AMP Study - NCT04431414: A Study of Immune Responses to the Virus That Causes COVID-19 |
| OT2HL156812 | - ACTIV Integration of Host-targeting Therapies for COVID-19 Administrative Coordinating Center | 498,112,068 | 9 & 200 | 0.04 | - NCT05023512: Understanding Public Attitudes Towards the COVID-19 Vaccination (CEAL) - NCT04355767: Convalescent Plasma in Outpatients With COVID-19 - NCT04505774: Accelerating COVID-19 Therapeutic Interventions and Vaccines 4 ACUTE |
| OT2HL161847 | - OTA-21-015A Post-Acute Sequelae of SARS-CoV-2 Infection Initiative: NYU Langone Health Clinical Science Core, Data Resource Core, and PASC Biorepository Core | 448,259,603 | - | - | - |
| UM1AI068636 | - Leadership and Operations Center (LOC), AIDS Clinical Trials Group (ACTG); LOC 1/ - AIDS Clinical Trials Group for Research on Therapeutics for HIV and Related Infections - CoVPN 3502 / ACTIV-2/A5401 - A Randomized, Double-Blind, Placebo-Controlled Trial to Evaluate the Efficacy of Hydroxychloroquine and Azithromycin to Prevent Hospitalization or Death in Persons with COVID-19 | 263,741,292 | - | - | - NCT04216589: Study of Semaglutide for Non-Alcoholic Fatty Liver Disease (NAFLD), a Metabolic Syndrome With Insulin Resistance, Increased Hepatic Lipids, and Increased Cardiovascular Disease Risk - NCT04636437: Doravirine for Persons With Excessive Weight Gain on Integrase Inhibitors and Tenofovir Alafenamide |
| UM1AI068619 | - CoVPN Vaccine Site Preparation - CoVPN 3008, Multi-Center, Randomized, Efficay Study of COVID-19 mRNA Vaccine in Regions with SARS-CoV-2 Variants of Concern - CoVPN 3501: Eli Lilly (LY3819253) Protocol Funding | 99,643,453 | - | - | - |
| U54HL143541 | - Clinical Studies Core - Center for Advancing Point of Care Technologies (CAPCaT) Administrative Supplement | 123,051,876 | 36 & 214 | 0.17 | - |
| U24TR001608 | - ACTIV-6 - COVID-19 Supplement - Center for Innovative TRIals in ChilDrEN and AdulTs (TRIDENT) | 116,412,856 | 13 & 117 | 0.10 | - NCT05736874: ACTIV-6: COVID-19 Study of Repurposed Medications - Arm C (Fluticasone) - NCT05890586: ACTIV-6: COVID-19 Study of Repurposed Medications - Arm B (Fluvoxamine) - NCT05736861: ACTIV-6: COVID-19 Study of Repurposed Medications - Arm A (Ivermectin 400) - NCT04885530: ACTIV-6: COVID-19 Study of Repurposed Medications |
| U24MD016258 | - RADx-UP CDCC - RADx-UP CDCC SAY YES COVID Test Study - RADx-UP SAY YES COVID Test Study (S4) | 69,559,557 | 2 & 0 | - | - NCT05212883: You and Me COVID Free - NCT05884515: Evaluation of Home Use COVID-19 Frequent Antigen Testing and Data Reporting |
| UM1AI068618 | - HVTN 405/HPTN 1901 Characterizing SARS-CoV-2-specific immunity in convalescent individuals: LC - CoVPN 3004 - A Phase 3, Randomizd, Observer-Blinded, Placebo-Controlled Study to Evaluate the Efficacy, Safety, and Immunogenicity of a SARS-CoV-2 Recombinant Spike Protein Nanoparticle Vaccine Lab - CoVPN 3002 A Phase III Randomized, Double-blind, Placebo-controlled Multicenter Study in Adults to Determine the Safety, Efficacy, and Immunogenicity of AZD1222 for the Prevention of COVID-19 LAB | 51,487,030 | 16 & 293 | 0.57 | - |
| OT2HL161841 | - Interactive Data Portals and Robust Analytic Tools to Wrap PASC Cohorts (iDRAW) OTA-21-015A | 51,373,541 | 1 & 0 | - | - |
| U54EB027690 | - Emergency COVID-19 supplement for Atlanta Center for Microsystems Engineered Point-of-Care Technoloites (ACME POCT) - Atlanta Center for Microsystems Engineered Point-of-Care Technologies (ACME POCT) | 49,359,942 | 14 & 49 | 0.10 | - |
| U54EB015408 | - Point of Care Technology Research Center in Primary Care | 34,989,814 | 5 & 7 | 0.02 | - |
| UL1TR002243 | - Passive Immunity Trial for Our Neighbors (PassITON): A randomized, placebo-controlled multi-site trial of anti-SARS-CoV-2 convalescent plasma to treat hospitalized adults with COVID-19 - Vanderbilt Institute for Clinical and Translational Research (VICTR) -Identifying correlates of functional immunity in SARS-CoV-2 convalescent plasma - Vanderbilt Institute for Clinical and Translational Research (VICTR) | 34,659,452 | - | - | - NCT04362176: Passive Immunity Trial for Our Nation to Treat COVID-19 in Hospitalized Adults |
| UL1TR001445 | - Clinical and Translational Science Award | 32,119,186 | - | - | - |
| UM1AI109565 | - Immune Tolerance Network | 31,078,250 | 34 & 507 | 1.63 | - NCT05027815: Tregs for the Treatment of Acute Respiratory Distress Syndrome (ARDS) Associated With COVID-19 (regARDS) - NCT03654040: Liver Transplantation With Tregs at UCSF - NCT04827979: Daratumumab and Belatacept for Desensitization - NCT04391309: COVID-19 and Anti-CD14 Treatment Trial |
| UM2AI117870 | - Rho Federal Systems Division, Inc. NIAID DAIT SACCC | 29,945,343 | 14 & 84 | 0.28 | - NCT03389893: Effect of Dupilumab (Anti-IL4Rα) on the Host-Microbe Interface in Atopic Dermatitis |
| U19AI110483 | - ACE Covid 19 Admin Supplement: Molecular Regulation of B cells and T cells in Human SLE - Molecular Regulation of B cells and T cells in Human SLE - Administrative Supplement Covid19: Molecular Regulation of B cells and T cells in Human SLE | 29,551,103 | 11 & 126 | 0.43 | - |
| UM1AI148689 | - Implementing Vaccine and Treatment Evaluation Unit (VTEU) Clinical Site:21-0011 - Vaccine Treatment Evaluation Units: Infectious Diseases Clinical Research Consortium - VTEU Supplement - Implementing Vaccine and Treatment Evaluation Unit (VTEU) Clinical Site - Vaccine Treatment Evaluation Units(VTEU) mRNA-1273-P204 Moderna Pediatric - Implementing Vaccine and Treatment Evaluation Unit (VTEU) Clinical Site: DMID 21-0012 Mix and Match - Implementing Vaccine and Treatment Evaluation Unit (VTEU) clinical site protocols - UMB Vaccine Treatment Evaluation Unit - DMID21-0004 - Implementing Vaccine and Treatment Evaluation Unit (VTEU) Clinical Site: CoVPN 3004 | 29,066,515 | 6 & 3226 | 11.10 | - |
| UM1AI148685 | - COVID-19 Supplement #3 Moderna Trial - Vaccine and Treatment Evaluation Unit at Saint Louis University - COVID-19 Supplement #5 for CoVPN Novavax Study - COVID-19 Supplement #4 for CoVPN 5002 Prevalence Study - Vaccine and Treatment Evaluation Unit at Saint Louis University - DMID 20-0034 | 25,775,548 | 1 & 0 | - | - |
| UM1AI148452 | - Vanderbilt Vaccine and Treatment Evaluation Unit - Vanderbilt Vaccine and Treatment Evaluation Unit – KidCOVE - Vanderbilt Vaccine and Treatment Evaluation Unit - DMID 21-0012 - Vanderbilt Vaccine and Treatment Evaluation Unit - DMID 21-0004 | 25,723,683 | 0 & 0 | - | - |
| S10AI160636 | - Personal Protective Equipment for Resources for COVID-19 Related Vaccine and Treatment Clinical Trials and Clinical Studies | 24,903,100 | 0 & 0 | - | - |
| UM1AI068635 | - CoVPN 3003 A Phase 3 Study to Assess the Efficacy and Safety of Ad26.COV2.S for the Prevention of SARS-CoV-2-mediated COVID-19 in Adults Aged 18 Years and Older SDMC - CoVPN 3006: A randomized controlled study to assess SARS-CoV-2 infection, viral shedding, and subsequent potential transmission in university students immunized with Moderna COVID-19 Vaccine - CoVPN Cross-Protocol Analyses - CoVPN 3001 A Phase 3, Randomized, Stratified, Observer-Blind, Placebo-Controlled Study to Evaluate the Efficacy, Safety, and Immunogenicity of mRNA-1273 SARS-CoV-2 Vaccine SDMC - CoVPN 5001 A prospective study of acute immune responses to SARS-CoV-2 infection SDMC - HVTN 405/HPTN 1901 Characterizing SARS-CoV-2-specific immunity in convalescent individuals | 24,803,421 | 73 & 790 | 3.19 | - |
| S10AI162059 | - Personal Protective Equipment for Resources for COVID-19 Related Vaccine and Treatment Clinical Trials and Clinical Studies | 21,360,749 | - | - | - |
| OT2HL158287 | - NHLBI Maternal Morbidity and Mortality (3M) Administrative Coordinating Center | 20,224,849 | - | - | - |
| UM1AI148575 | - Vaccine and Treatment Evaluation Units (VTEU) - Vaccine and Treatment Evaluation Units (VTEU): CoVPN 5002: SARS-CoV-2 Prevalence Study - Vaccine and Treatment Evaluation Units (VTEU)-DMID 21-0004 | 19,003,792 | - | - | - |
